# Successful thoracoscopic evacuation of an extrapleural hematoma with delayed symptomatic pleural effusion: a case report

**DOI:** 10.1186/s40792-019-0691-9

**Published:** 2019-08-14

**Authors:** Masanori Shimomura, Shunta Ishihara, Masashi Iwasaki, Masayoshi Inoue

**Affiliations:** 1 Division of Thoracic Surgery, Department of Surgery, Kyoto Prefectural University of Medicine, Kyoto, 602-8566 Japan; 2 Department of General Thoracic Surgery, Ayabe City Hospital, Ayabe, Japan

**Keywords:** Chest wall, Pleura, Trauma, Blunt

## Abstract

**Background:**

Traumatic extrapleural hematoma is a rare condition and is usually managed conservatively until spontaneous resolution unless active bleeding or expansion is found.

**Case presentation:**

An 80-year-old man taking an anticoagulant medication was referred to our hospital after accidentally falling in a street ditch while riding a bike. Chest X-ray and computed tomography (CT) scan showed multiple fractures on ribs 7–9, hemothorax, and extrapleural hematoma in the posterior chest wall. Though the patient’s hemothorax was improved by chest tube drainage, the extrapleural hematoma still remained. He was transferred to another hospital for rehabilitation, but he was readmitted to our hospital because of dyspnea with accumulation of left pleural effusion, including a subpopulation of neutrophils, but without bacterial infection. We performed thoracoscopic evacuation of the hematoma on day 57 after the initial blunt chest trauma. The patient has had no recurrence of pleuritis for 6 months after surgery.

**Conclusion:**

Since posttraumatic extrapleural hematoma may result in delayed secondary intractable pleural effusion causing dyspnea, careful observation is necessary when considering indications of surgical intervention.

## Background

An extrapleural hematoma is a rare and occasionally life-threatening condition defined as hemorrhage between the parietal pleura and endothoracic fascia. This condition may occur as a complication after blunt chest trauma associated with rib or sternal fractures, which cause injury to the intercostal or parasternal vessels. In the acute phase, patients with extrapleural hematomas should be treated surgically if their vital signs are unstable or if they are in hypovolemic shock. Otherwise, the patient’s status can be observed for active bleeding or expansion of the hematoma until spontaneous resolution. We herein present the case of a patient with multiple rib fractures and an extrapleural hematoma requiring surgical treatment due to a delayed intractable pleural effusion during the chronic phase.

## Case presentation

**Fig. 1:**
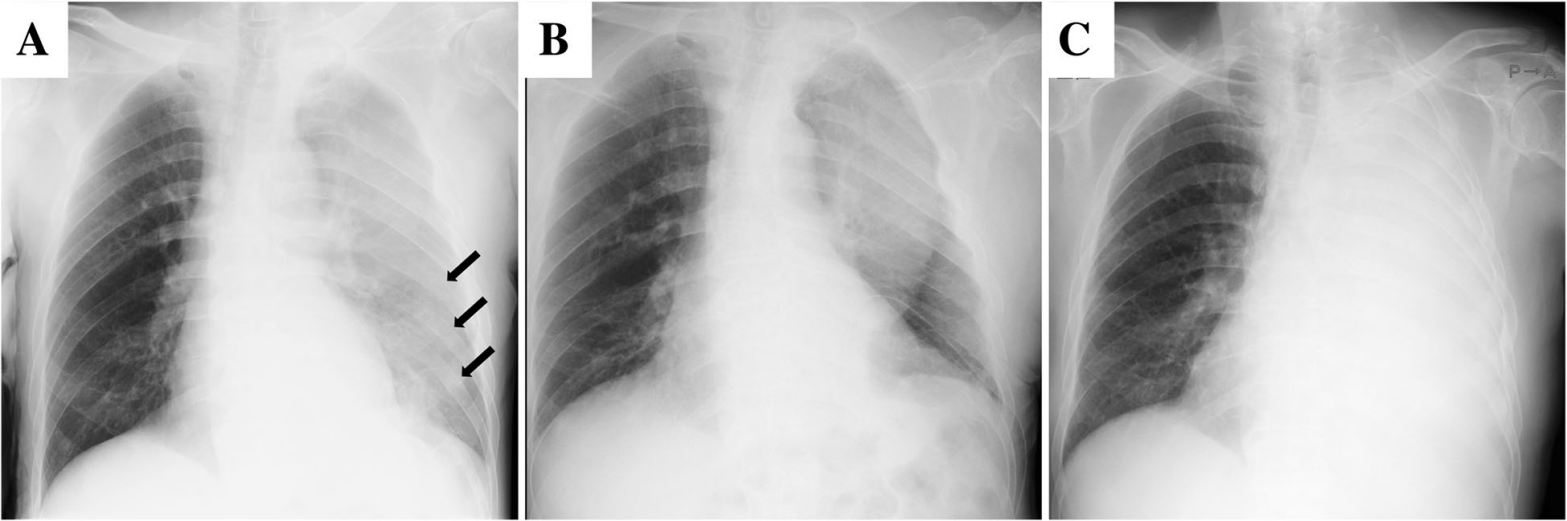
Chest radiograph showing the left multiple rib fractures (black arrow), a hemothorax, and an extrapleural hematoma on admission (**a**); improved hemothorax with the residual extrapleural hematoma 5 days after the episode of trauma (**b**); and a large amount of pleural effusion 53 days after the episode of trauma (**c**)

**Fig. 2:**
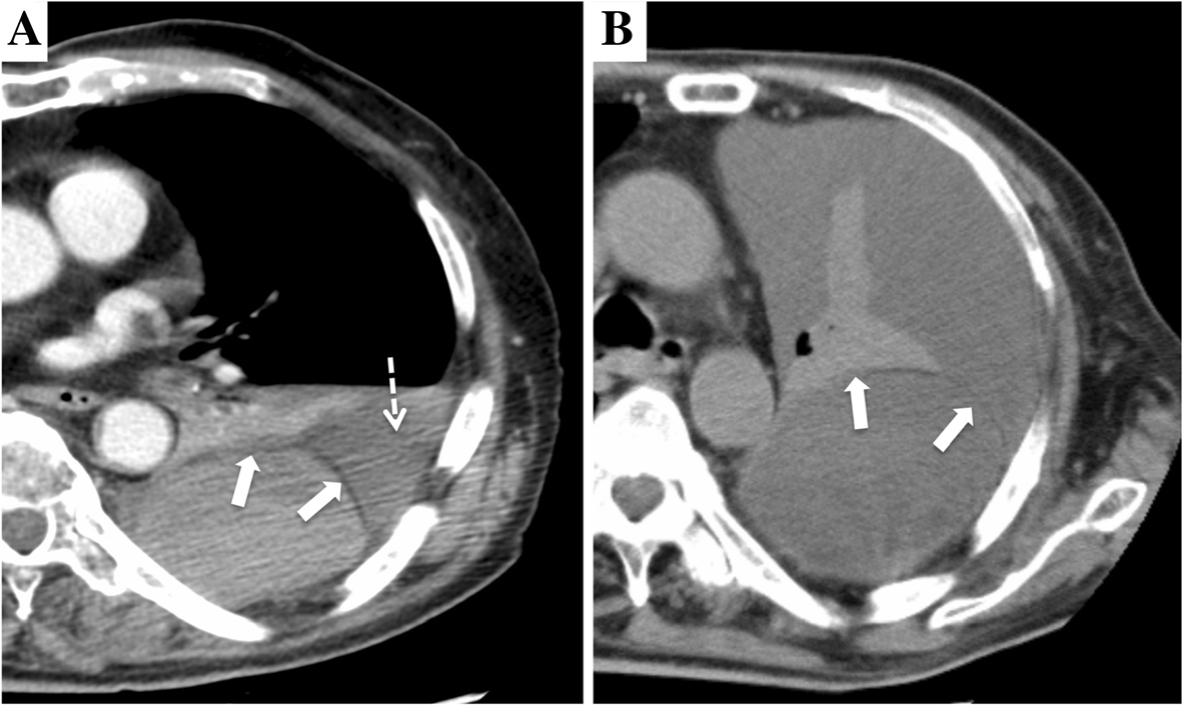
Chest computed tomography showing hemothorax (dashed arrow), a convex extrapleural hematoma with extrapleural fat sign (white arrow), and atelectasis of the left lower lobe on the patient’s posterior chest wall on admission (**a**) and a large amount of pleural effusion and atelectasis with a residual extrapleural hematoma (white arrow) 53 days after the episode of trauma (**b**)

**Fig. 3:**
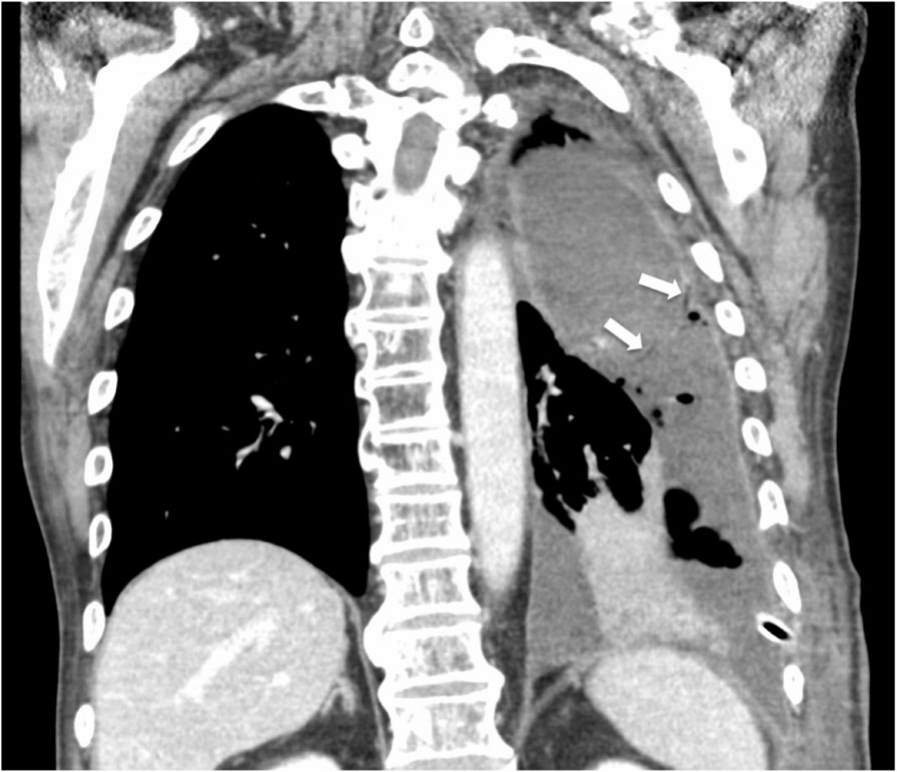
Preoperative chest computed tomography showing poor lung re-expansion, loculated pleural effusions, and a residual extrapleural hematoma with extrapleural fat signal (white arrow) after placement of the second chest tube

An 80-year-old man was referred to our hospital because of an accidental fall into a street ditch when he was riding a bike. The patient was alert and oriented, and his vital signs were stable: heart rate of 90 beats/min, blood pressure of 142/68 mmHg, and SpO2 of 96% (room air). A chest X-ray and computed tomography (CT) scan showed left 5th, 7th, 8th, and 9th rib fractures; fractures of the left scapula and L1 transverse process; a hemothorax; and a biconvex extrapleural hematoma measuring 9.1 cm in diameter in the posterior chest wall (Figs. 1a and 2a). Neither extravasation nor active bleeding of the intercostal artery or vessels around the scapula was detected. The patient had a past history of acute coronary syndrome 5 years previously, which was treated with coronary stent implantation followed by medical treatment with aspirin and prasugrel hydrochloride. An echocardiogram demonstrated normal to mildly decreased left ventricular systolic function. He also had been on insulin for diabetes. Regarding his present admission, a total volume of 1000 ml of bloody pleural effusion was drained immediately after the placement of a chest tube. Fortunately, the volume of intrathoracic hemorrhage gradually decreased, and the patient was managed conservatively with a transfusion. The chest tube was removed 5 days later when the amount of drainage decreased to less than 200 ml/day. The extrapleural hematoma, however, still remained in the posterior chest wall as visualized on a chest X-ray (Fig. 1b). The pleural effusion did not increase remarkably on X-ray after removal of the chest tube. Thus, we anticipated the spontaneous resolution of the extrapleural hematoma and pleural effusion because the patient had maintained a stable condition in terms of his vital signs and respiratory status, so he was transferred to a neighboring hospital for rehabilitation 27 days after the episode of trauma. However, he developed dyspnea due to the accumulation of a left pleural effusion 53 days after the episode trauma (Figs. 1a and 2b). A chest tube was placed again to drain a large amount of serous effusion, which contained neutrophils without bacterial infection. A chest CT acquired after placement of the chest tube showed a residual extrapleural hematoma and multiloculated pleural effusions on the diaphragm (Fig. 3). Thus, we considered that the effusion might have been influenced by an inflammatory reaction due to the residual extrapleural hematoma. At 57 days after the episode of trauma, we underwent thoracoscopic evacuation of the hematoma through a 5-cm mini-thoracotomy in the eighth intercostal space along the posterior axillary line with an incision for thoracoscopy in the sixth intercostal space along the anterior axillary line. The reason that we used a mini-thoracotomy was to prepare for any difficulties we might encounter during the operation because we expected the hematoma to be rigid or organized and therefore not only evacuation but also debridement to be required.

**Fig. 4:**
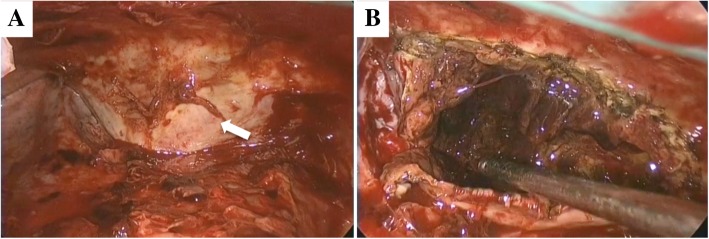
Intraoperative findings of a pleural bulge in the posterior chest wall due to the extrapleural hematoma (white arrow) (**a**) and dissection of the thick dorsal parietal pleura to resect a large amount of hematoma (**b**)

We found thick parietal pleura in the overall thoracic space and a large pleural bulge in the posterior chest wall due to the extrapleural hematoma (Fig. 4a). We dissected the thick dorsal parietal pleura to evacuate the hematoma and irrigate the space (Fig. 4b). There was no active hemorrhage in the extrapleural space during surgery. We placed chest tubes in both the thoracic space and the extrapleural space. The chest tubes were successfully removed on the 7th postoperative day, and the patient was discharged on the 10th postoperative day. The patient has had no recurrence of pleuritis for 6 months.

## Discussion

Extrapleural hematomas occur in approximately 7% of patients with blunt chest injuries [1]. These patients almost always have complications of rib fractures and hemothorax [1, 2]. Hemothorax after thoracic trauma can result in serious conditions, such as hemorrhagic shock. Conversely, the extrapleural space is a rare site of blood pooling because of the limited space between the parietal pleura and thoracic muscle fascia. Usually, a watch-and-wait approach can be taken if the vital signs of the patient are stable, there is no progression of anemia, and the hematoma does not expand in size. Several cases of conservative treatment for extrapleural hematomas including observation or image-guided drainage have been reported [3, 4]. Regarding the indications for surgical treatment, Chung et al. reported that a biconvex extrapleural hematoma on CT tended to be larger than other types (nonconvex), and surgical intervention might be necessary [2]. Rashid et al. also reported a single case of a patient who underwent surgical treatment with thoracotomy following insufficient needle aspiration drainage, while conservative treatment with observation and chest X-ray monitoring was performed in the remaining 33 patients with extrapleural hematomas [1]. However, patients with extrapleural hematomas who are administered antithrombotic or anticoagulant agents should be strictly followed up because they have a high risk of rapid expansion of the hematomas [5, 6]. Posttraumatic pleural effusions might be caused by the infiltration of eosinophils into the pleural space [7] or bacterial empyema [8]. In the present case, a large amount of pleural effusion was found in the left thoracic cavity with a residual extrapleural hematoma 2 months after the patient sustained a chest injury. With neither infection nor eosinophilic infiltration in the pleural effusion and the thickened parietal pleura as intraoperative findings, we speculated that a relatively large chronic hematoma outside the parietal pleura contributed to secondary pleural inflammation and resulted in delayed pleuritis.

## Conclusions

Residual extrapleural hematoma occupying a portion of the thoracic cavity should be carefully observed in patients undergoing conservative treatment, and surgical resection might be an option for nonregressive lesions.

## Data Availability

The dataset supporting the conclusion of this article is included within the article.

## Abbreviation

CT: Computed tomography
